# A double-blind, placebo-controlled trial of the efficacy and safety of two doses of azelastine hydrochloride in perennial allergic rhinitis

**DOI:** 10.3389/falgy.2023.1244012

**Published:** 2023-10-18

**Authors:** Jean Bousquet, Ludger Klimek, Hans-Christian Kuhl, Duc Tung Nguyen, Rajesh Kumar Ramalingam, G. W. Canonica, William E. Berger

**Affiliations:** ^1^ Institute of Allergology, Charité – Universitätsmedizin Berlin, Corporate Member of Freie Universität Berlin and Humboldt-Universität zu Berlin, Berlin, Germany; ^2^ Allergology and Immunology, Fraunhofer Institute for Translational Medicine and Pharmacology ITMP, Berlin, Germany; ^3^ Center for Rhinology and Allergology, Wiesbaden, Hessen, Germany; ^4^ Biometrics, Meda Pharma GmbH & Co KG (A Viatris Company), Bad-Homburg, Germany; ^5^ Global Clinical Sciences, MEDA Pharma GmbH & Co KG (A Viatris Company), Bad Homburg, Germany; ^6^ Mylan Pharmaceuticals Private Limited (Now Viatris), Bengaluru, India; ^7^ Department of Biomedical Sciences, Humanitas University, Milan, Italy; ^8^ Asthma & Allergy Unit, IRCCS Humanitas Research Hospital, Milan, Italy; ^9^ Allergy & Asthma Solutions, Inc., Coto de Caza, CA, USA

**Keywords:** perennial allergic rhinitis, azelastine hydrochloride, intranasal antihistamines, allergic rhinitis, randomized clinical trial

## Abstract

**Background:**

Azelastine hydrochloride (AZE) is a selective, non-sedating H1 antagonist with anti-inflammatory and mast cell stabilizing properties, which can be used as an alternative to intranasal corticosteroids. The objective of this study was to evaluate the efficacy of the new formulation of 0.15% AZE compared to that of the placebo at a dosage of two sprays per nostril twice daily for 4 weeks in patients with perennial allergic rhinitis (PAR).

**Materials and methods:**

A total of 581 subjects were randomized in this double-blind (DB) placebo-controlled trial (NCT00712920) that compared 0.10% (1,096 μg daily) and 0.15% AZE (1,644 μg daily) to the placebo in PAR patients. The study consisted of a 7-day single-blind placebo lead-in period and a 28-day DB treatment period. The primary endpoint was the change from baseline in the 12-h reflective total nasal symptom score (rTNSS) for the entire 28-day study period of 0.15% AZE, two sprays per nostril BID compared to the placebo. The efficacy and safety of 0.15% AZE were compared to the placebo.

**Results:**

Least square (LS) mean improvement from baseline in the morning (AM) and evening (PM) combined rTNSS was statistically significant for the 0.15% AZE group (*p* = 0.04) compared to the placebo group. LS mean improvement from baseline in the AM and PM combined rTNSS was 4.10 (4.26) units for 0.15% AZE and 3.81 (3.99) for 0.10% AZE. For individual symptoms, there was a statistically significant change in the LS mean (*p* = 0.04) improvement from baseline on the 12-h reflective assessment for the 0.15% AZE group for runny nose. Further numerical improvements were shown for itchy nose, nasal congestion, runny nose, and sneezing compared to the placebo. No deaths or serious adverse events related to the study medication were reported.

**Conclusion:**

The present formulation of 0.15% AZE is safe and effective in relieving PAR symptoms. It effectively relieves nasal and non-nasal symptoms.

**Clinical Trial Registration:**

ClinicalTrials.gov, identifier: NCT00712920.

## 1. Introduction

Traditionally, allergic rhinitis (AR) is distinguished into two forms: seasonal AR (SAR, triggered by outdoor allergens) and perennial AR (PAR, triggered by indoor allergens). Existing monotherapies such as oral and intranasal antihistamines, intranasal corticosteroids (INCS), oral and intranasal decongestants, and oral and intranasal anticholinergics are not sufficient to control AR in most patients. Patients are hesitant to use INCS due to concerns about the potential systemic effects of corticosteroids (1). Improving the patient's knowledge about corticosteroids also fails to reduce steroid phobia (2).

Azelastine hydrochloride (AZE) is a selective, non-sedating H_1_ antagonist with anti-inflammatory and mast cell-stabilizing properties (3). AZE is an alternative to corticosteroids and is available as both nasal spray and eye drops. It has similar efficacy to intranasal steroids (4) but a faster onset of action (≤15 min) (5). AZE is recommended for treating SAR and PAR symptoms such as rhinorrhea, sneezing, and nasal pruritus in adults and children ≥5 years of age. JTF practice parameters and guidelines 2020 also recommend using AZE as a first line of treatment for AR (6). A well-established formulation of 0.10% AZE has been found to be effective and safe for treating SAR and PAR in adults and children ≥5 years of age (7). Studies with concentrations of 0.15% AZE have shown greater dose-dependent efficacy in controlling SAR compared to 0.10% AZE while maintaining safety and tolerability (8–10). The daily applied dose of 0.10% AZE, according to the label, is 1,096 μg. The daily applied dose of 0.15% AZE is 822 μg when administered as two sprays per nostril OD and 1,644 μg when administered as two sprays per nostril BID. Viatris Inc. has developed a new formulation of 0.15% AZE with sorbitol and sucralose to mask the bitter taste of the original formulations.

This study aimed to evaluate the efficacy of the new formulation of 0.15% AZE compared to that of the placebo at a dosage of two sprays per nostril twice daily for 4 weeks in patients with PAR. The efficacy and safety of 0.15% AZE was also compared to those of 0.10% AZE solution and placebo.

## 2. Methods

### 2.1. Trial design

**Figure 1:**
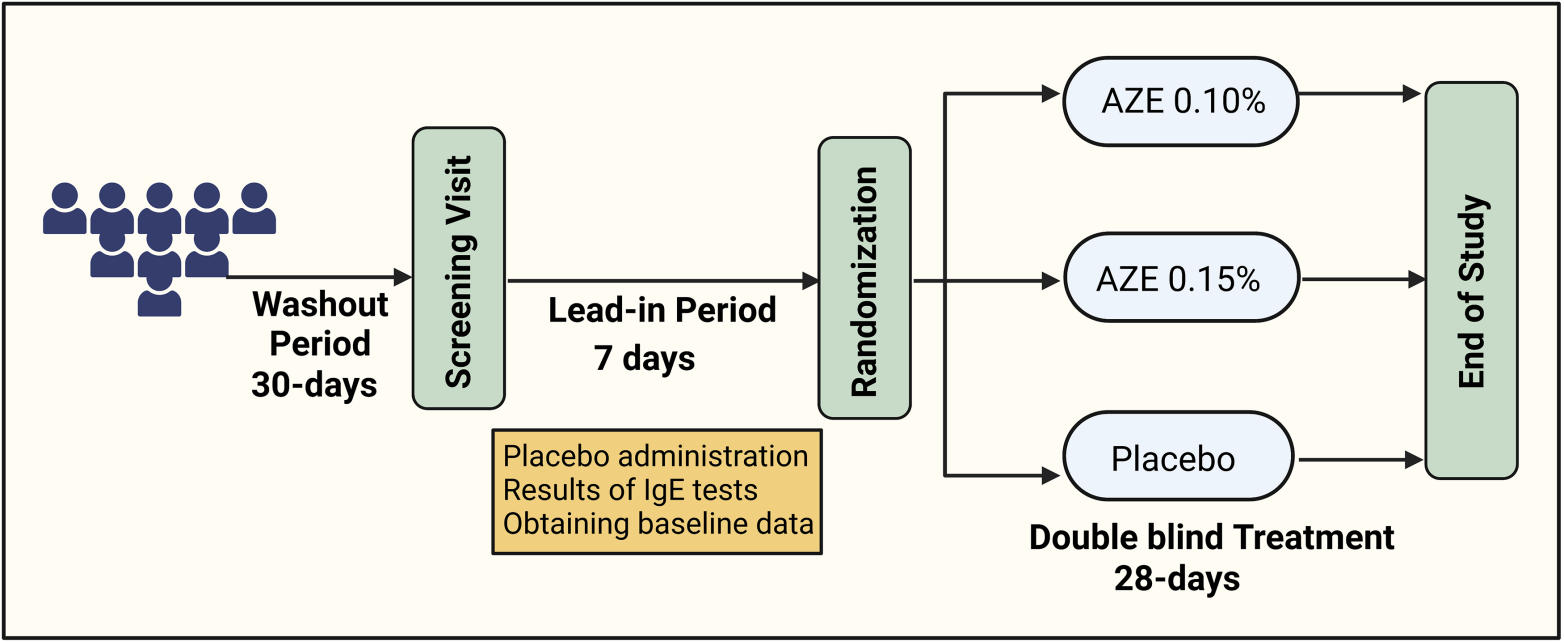
Flowchart of the study design (created using biorender.com).

This was a randomized, double-blind, placebo-controlled trial (DB-PC-RCT) that compared 0.10% AZE (1,096 μg daily) and 0.15% AZE (1,644 μg daily) to a placebo in PAR patients (Figure 1). The allocation ratio was 1:1:1. The study consisted of a 7-day single-blind placebo lead-in period and a 28-day DB treatment period. The primary endpoint was the change from baseline in the 12-h reflective total nasal symptom score (rTNSS) for the entire 28-day study period of 0.15% AZE two sprays per nostril BID compared to the placebo. The efficacy and safety of 0.15% AZE were compared to those of the placebo. Secondary endpoints included several outcomes (Table 1).

**Table 1:** Primary and secondary efficacy endpoints.

Primary efficacy endpoint	Secondary efficacy endpoints
(i) Change from baseline in the 12-h rTNSS for the entire 28-day study period compared to the placebo	(i) Change from baseline in 12-h rTNSS individual symptom scores for the entire 28-day study period compared to the placebo (ii) Change from baseline in 12-h rSSCSs (consisting of postnasal drip, itchy eyes, cough, and headache) for the entire 28-day study period compared to the placebo (iii) Change from baseline in 12-h rSSCS individual symptom scores for the entire 28-day study period compared to the placebo

### 2.2. Settings

In total, 43 investigational sites in the U.S. were selected from February to October 2007, prior to the onset of the pollen season to avoid interference with symptoms of seasonal allergic rhinitis (SAR) due to pollen.

### 2.3. Participants

Patients aged over 12 years of age with a diagnosis of allergic rhinitis, a history of symptoms for more than 2 years, and a positive skin prick test to one or more of the allergens such as dust mites, cockroach, mold, and cat or dog dander during the previous year were eligible for randomization. IgE-mediated hypersensitivity to dust mites, cockroaches, mold, and cat or dog dander was confirmed by a positive response to a skin prick test within the previous year. A positive response was defined as a wheal diameter at least 3 mm greater than the negative control in the skin prick test. Patients receiving immunotherapy injections (antigen desensitization) had to be on a stable maintenance regimen for at least 30 prior to the first study visit. Concomitant medications that could confound study results were discontinued before enrollment, and their use was prohibited during the study period. The following washout periods are generally sufficient as per FDA guidelines: (i) intranasal or systemic corticosteroids (1 month); (ii) leukotriene modifiers (1 month); (iii) intranasal cromolyn (2 weeks); (iv) intranasal or systemic decongestants (3 days); (v) cetirizine, fexofenadine, loratadine, desloratadine, hydroxyzine (5–10 days); (vi) intranasal antihistamines (3 days); and (vii) other systemic antihistamines (3 days).

The patients had to have moderate/severe PAR symptoms. At screening, subjects with a 12-h rTNSS [morning (AM) or evening (PM)] of at least 6/12 and a congestion score of 2–3/3 were eligible for inclusion. At inclusion, subjects were randomized if they had a 12-h rTNSS (AM or PM) of at least six on three separate symptom assessments (one of which was within 2 days of randomization) and a 12-h rTNSS of 2–3/3 separate symptom assessments (one of which was within 2 days of day 1).

Key exclusion criteria included (i) the presence of nasal ulceration or nasal septal perforation, (ii) nasal surgery/sinus surgery within the previous year, (iii) pulmonary disease like asthma, (iv) the presence of clinically significant nasal polyposis /nasal structural abnormalities, (v) pregnant or nursing women, and (vi) hypersensitivity to drugs similar to azelastine and either sorbitol or sucralose.

### 2.4. Ethics

The study was reviewed and approved by institutional review boards (IRBs) at the participating sites. The Ethics Board was the Sterling Institutional Review Board, with approval numbers 2397-001 through 2397-043 (for 43 study sites). Patients provided written informed consent/pediatric assent. If the patient was a minor, written informed consent was obtained from a parent or legal guardian. The study followed all the applicable laws and guidelines of the International Conference on Harmonization (ICH)/Good Clinical Practices (GCP), following the principles of the Declaration of Helsinki, and the U.S. Code of Federal Regulations (CFR).

### 2.5. Outcomes

During the treatment period, patients recorded the four nasal symptoms (runny nose, nasal congestion, itchy nose, and sneezing) in a diary prior to the AM and PM doses of the study medication on each study day. The severity of the symptoms was scored on a four-point scale (0–3), where 0 = no symptoms, 1 = mild symptoms, 2 = moderate symptoms, and 3 = severe symptoms. The rTNSS is the sum of the scores for each of the nasal symptoms (from 0 to 12) assessed twice daily (0–24). The 12-h reflective secondary symptom complex score (SSCS), comprising intensity scores for postnasal drip, itchy eyes, cough, and headache, was also measured.

### 2.6. Randomization and blinding

Once a potential study subject was identified and informed consent was signed, the subject was enrolled into the study and assigned a unique identification number consisting of two parts, the study site number and the subject number. Subject identification numbers were assigned sequentially in ascending order within each study site by the investigator. The subject identification number was used on all case report form, diaries, study medication supplies, and drug assignment records. Once issued, subject identification numbers were not reused and remained with the subject throughout the study. Randomization codes were provided by i3 Statprobe using a validated system that assigns random permutations of treatments to consecutive groups of six subjects. Randomization data were kept confidential.

On day 1, subjects received blinded study medication according to the randomization schedule. The study blinding was preserved at the study sites until all subjects had completed the trial and the database had been locked, except for individual subject data in the context of a serious safety concern. Codebreaker labels were detached from the study medication kits and affixed to the drug assignment record. A codebreaker label was to be opened by the investigator only in case of a medical emergency [e.g., a serious adverse event (SAE)] when it was judged necessary to know a subject's treatment assignment).

### 2.7. Sample size calculation

Based on a previous study with 0.10% AZE in patients with SAR and considering a reduction of 1.5 units in AM and PM combined TNSS with a standard deviation (SD) of 4.8, it was determined that a sample size of approximately 180 patients per treatment group was required to demonstrate efficacy with two sprays per nostril twice daily compared to the placebo in the 0.10% AZE group and to demonstrate an observable dose–response difference between 0.10% AZE and 0.15% AZE. Since the effect size with two sprays per nostril twice daily of 0.15% AZE was greater than that of 0.10% AZE, the sample size of 180 patients per group was adequate to fulfill the primary objective of showing a statistically significant reduction in the overall change from baseline in the 12-h rTNSS at the 0.05 level of significance with 80% power.

A gatekeeping strategy was employed to adjust for multiplicity. The 0.15% AZE–placebo comparison was first tested at the 0.05 significance level. If significant, the 0.10% AZE–placebo comparison was also performed at the 0.05 level. If the 0.15% AZE–placebo comparison was not significant at the 0.05 level, no comparison of the low dose of AZE to the placebo was made. The multiplicity strategy was limited to the primary analysis, and from a formal perspective, a secondary analysis was regarded as exploratory.

## 3. Statistical analyses

For continuous variables like TNSS, descriptive statistics included the number of patients reflected in the calculation (*n*), mean, standard deviation, median, minimum, and maximum. Categorical data were expressed as frequencies and percentages. A repeated-measures analysis was performed on the primary efficacy variable, change from baseline in the 12-h rTNSS, for the entire 28-day study period, compared to the placebo, which included all changes in the rTNSS on each day from day 1 PM to day 28 AM as repeated measures in an analysis of covariance (ANCOVA) model for the intent-to-treat (ITT) population, defined as all randomized subjects who had at least one postbaseline efficacy observation. The model contained study day as a within-subject effect, treatment group, and site as the between-subject effects, and baseline as a covariate.

Except for further efficacy analyses of the primary endpoint results, all other secondary efficacy analyses were performed as described for the primary efficacy endpoint. Only the ITT population was used for the change from baseline to day 28 for the overall score. Safety analyses were performed on the safety population, defined as all randomized subjects who received at least one dose of study medication. The incidence of treatment-related AEs was summarized by the body system and preferred term for overall AEs and by maximum severity and relationship to the study drug. All statistical conclusions were based on a 0.05 level of significance. All statistical tests were two-sided.

### 3.1. Handling of missing data and dropouts

The last observation carried forward (LOCF) method was used for dropouts and missing data. If a postbaseline TNSS score was missing, the last non-missing postbaseline TNSS score prior to the missing value was used for analysis. The same methodology was applied to the SSCS assessments. The LOCF method was also applied for summaries of individual nasal symptom scores and individual secondary symptom scores.

## 4. Results

### 4.1. Demographic characteristics of the patients

**Figure 2:**
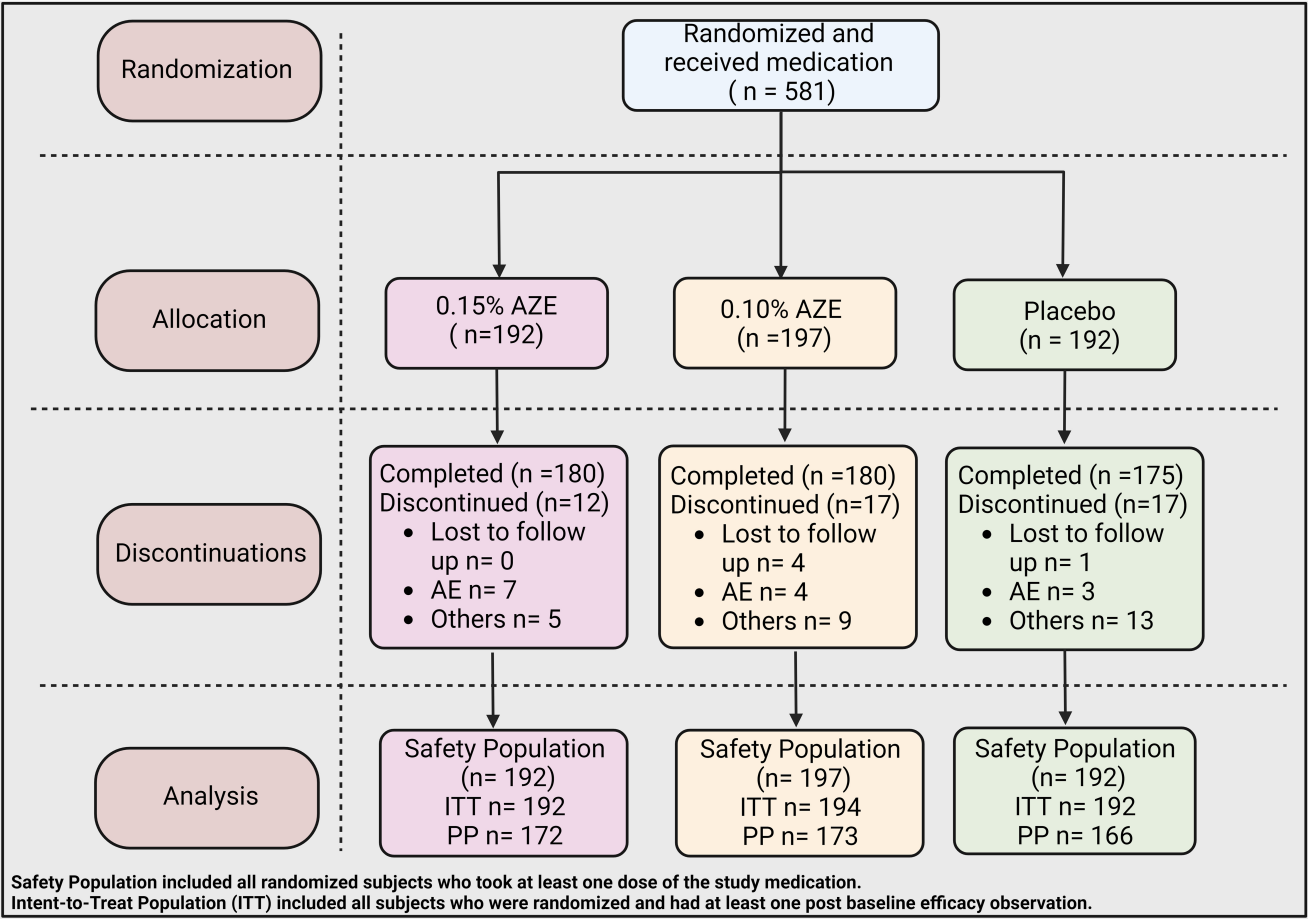
CONSORT flow diagram of the treatment procedure.

A total of 581 subjects were randomized into the study, of which 535 (92.1%) completed the study (Table 2 and Figure 2). Completion rates were similar across the three treatment groups (AZE-0.15, AZE-0.10, and placebo). The primary cause of discontinuation was due to “other” reasons (e.g., early onset of ragweed season, jury duty, moving, etc.). The ITT set had 578 subjects, whereas the PP (per protocol) population had 511.

**Table 2:** Demographic characteristics of the ITT population participants.

	0.15% AZE	0.10% AZE	Placebo
Demographics			
Age (years)			
Mean (SD)	36.9 (13.09)	35.6 (13.31)	38.1 (15.37)
Gender (*N* %)			
Male	58 (29.9)	65 (33.9)	62 (32.3)
Female	136 (70.1)	127 (66.1)	130 (67.7)
Ethnicity (*N* %)			
Hispanic or Latino	32 (16.5)	36 (18.8)	29 (15.1)
Not Hispanic or Latino	162 (83.5)	156 (81.3)	163 (84.9)
Race (*N* %)			
Black	28 (14.4)	26 (13.5)	11 (5.7)
White	160 (82.5)	159 (82.8)	172 (89.6)
Height (in.)			
Mean (SD)	65.8 (3.68)	65.9 (4.22)	66.1 (4.18)
Weight (lb.)			
Mean (SD)	169.4 (37.01)	175.2 (46.70)	177.9 (47.13)
Baseline characteristics			
Total TNSS score			
Mean (SD)	15.9 (3.8)	15.6 (3.89)	14.8 (3.99)
Duration of PAR history (years)			
Mean (SD)	19.0 (12.69)	12.82 (17.0)	13.45 (17.0)

The three treatment groups were comparable with regard to demographics (Table 1). The baseline TNSS scores for the three groups were 15.9 (AZE-0.15), 15.6 (AZE-0.10), and 14.8 (placebo). There was a difference in the baseline TNSS scores among the three groups (*p* = 0.017). The compliance to the study drug ranged from 90.1% (placebo) to 92.9% (AZE-0.15).

### 4.2. Primary endpoint

**Figure 3:**
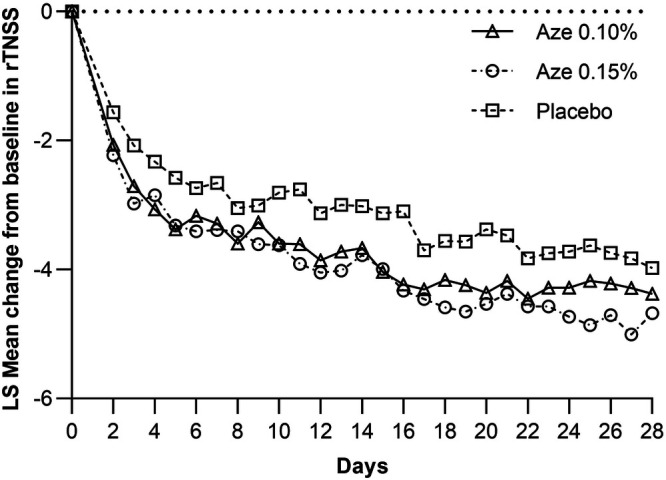
Plot of mean change from baseline in the rTNSS: AM and PM.

Least square (LS) mean improvement from baseline in the AM and PM combined rTNSS was statistically significant for 0.15% AZE (*p* = 0.04) compared to the placebo group (Figure 3). LS mean improvement from baseline in the AM and PM combined rTNSS was 4.10 (4.26) units for 0.15% AZE and 3.81 (3.99) units for 0.10% AZE. The 0.15% AZE dose showed a numerically greater improvement in the AM and PM combined rTNSS than 0.10% AZE. The difference between the 0.10% AZE and placebo groups was not statistically significant (*p* = 0.15). In placebo-treated patients, rTNSS scores decreased by 3.33 (4.35) units at 4 weeks.

### 4.3. Secondary endpoints

For individual symptoms, there was a statistically significant change in the LS mean (*p* = 0.04) and the LS mean percentage (*p* = 0.04) from baseline on the 12-h reflective assessment for the 0.15% AZE group for runny nose. Further numerical improvements were shown for itchy nose, nasal congestion, runny nose, and sneezing compared to the placebo (Table 3).

**Table 3:** Change from baseline in the reflective individual nasal symptom score: AM and PM combined (intent-to-treat population).

Treatment groups	LS mean baseline (SD)	LS mean change from baseline (SD)	*P*-value vs. placebo (95% CI)
Itchy nose			
0.10% AZE	3.86 (1.392)	−0.88 (1.135)	0.552 (−0.30 to 0.16)
0.15% AZE	3.95 (1.393)	−0.93 (1.342)	0.299 (−0.36 to 0.11)
Placebo	3.68 (1.374)	−0.81 (1.310)	
Nasal congestion			
0.10% AZE	4.71 (0.929)	−0.99 (1.253)	0.520 (−0.31 to 0.15)
0.15% AZE	4.76 (0.958)	−1.03 (1.228)	0.317 (−0.35 to 0.11)
Placebo	4.62 (0.986)	−0.92 (1.197)	
Runny nose			
0.10% AZE	3.82 (1.337)	−1.01 (1.239)	0.375 (−0.35 to 0.13)
0.15% AZE	3.92 (1.290)	−1.16 (1.321)	0.039 (−0.49 to −0.01)
Placebo	3.66 (1.350)	−0.91 (1.418)	
Sneezing			
0.10% AZE	3.26 (1.462)	−0.91 (1.239)	0.130 (−0.41 to 0.05)
0.15% AZE	3.30 (1.493)	−0.95 (1.339)	0.065 (−0.45 to 0.01)
Placebo	2.90 (1.502)	−0.74 (1.207)	

**Figure 4:**
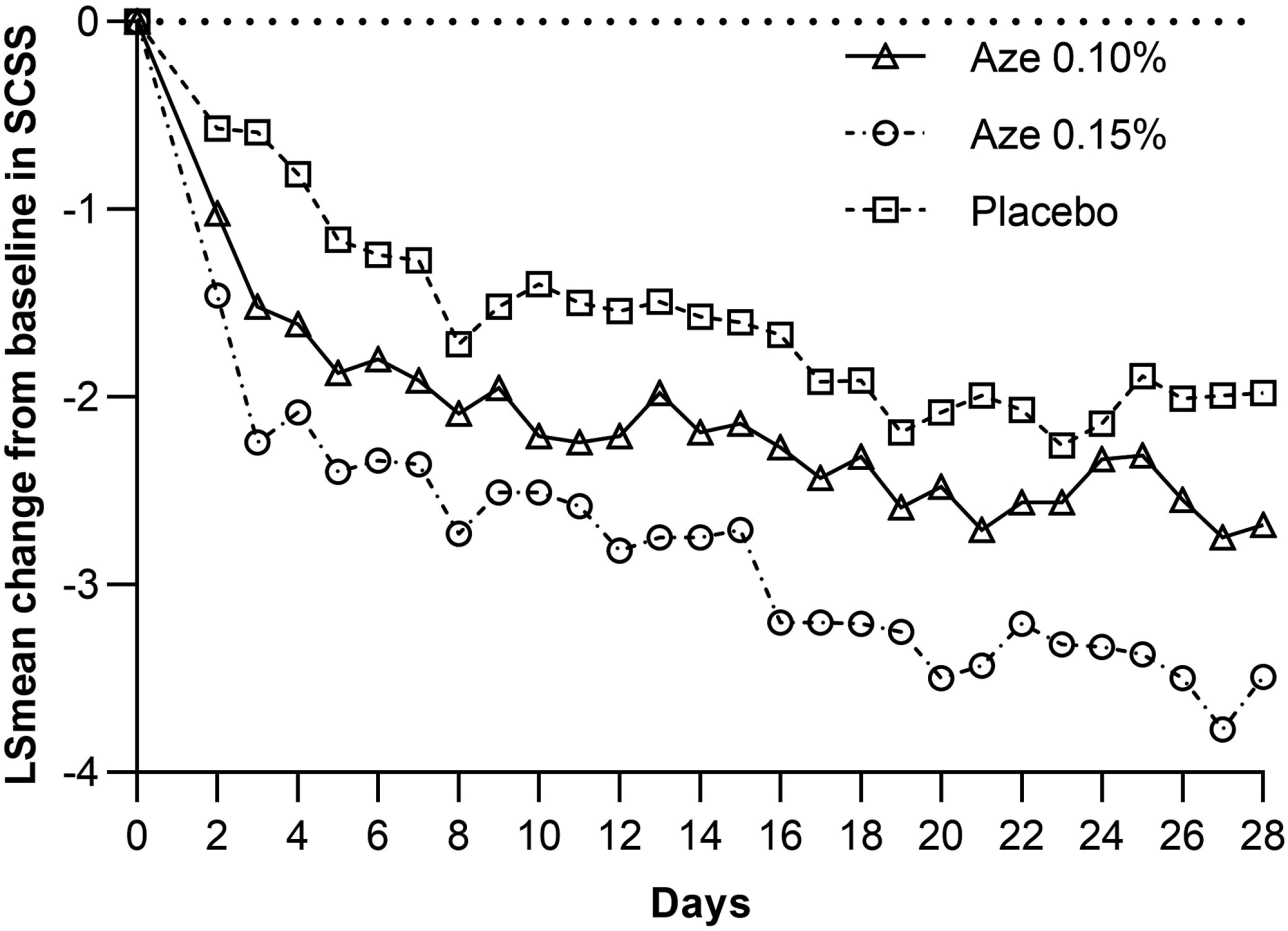
LS mean change from baseline in total rSSCS.

The LS mean change from baseline in the overall reflective secondary symptom complex scores (rSSCSs) showed a statistically significant improvement for the 0.15% AZE group (−2.92) compared to the placebo group (−1.79) after 4 weeks of treatment (*p* = 0.002). This was however not confirmed for 0.10% AZE (*p* > 0.05) (Table 4 and Figure 4). The LS mean change from baseline in the 12-h rSSCS for itchy eyes (*p* < 0.001), cough (*p* = 0.028), and headache (*p* = 0.008) demonstrated statistically significant improvements for the 0.15% AZE group compared to the placebo. The difference was also greater for the 0.10% AZE group compared to the placebo (*p* < 0.053).

**Table 4:** Change from baseline in the reflective individual symptom score: AM and PM combined (intent-to-treat population).

Treatment groups	LS mean baseline (SD)	LS mean change from baseline (SD)	*P*-value vs. placebo (95% CI)
Postnasal drip			
0.10% AZE	4.09 (1.499)	−0.76 (1.336)	0.911 (−0.26 to 0.23)
0.15% AZE	4.22 (1.455)	−0.88 (1.294)	0.288 (−0.38 to 0.11)
Placebo	4.02 (1.569)	−0.74 (1.297)	
Itchy eyes			
0.10% AZE	3.40 (1.701)	−0.62 (1.265)	0.053 (−0.46 to 0.00)
0.15% AZE	3.55 (1.604)	−0.80 (1.294)	<0.001 (−0.64 to −0.17)
Placebo	3.14 (1.706)	−0.39 (1.295)	
Cough			
0.10% AZE	2.14 (1.732)	−0.49 (1.419)	0.567 (−0.29 to 0.16)
0.15% AZE	2.38 (1.715)	−0.68 (1.344)	0.028 (−0.48 to 0.03)
Placebo	2.13 (1.548)	−0.91 (1.418)	
Headache			
0.10% AZE	2.15 (1.836)	−0.38 (1.175)	0.295 (−0.32 to 0.10)
0.15% AZE	2.25 (1.784)	−0.55 (1.313)	0.008 (−0.49 to −0.07)
Placebo	1.90 (1.837)	−0.74 (1.207)	

### 4.4. Safety

The mean duration of exposure was 27.4 days for patients in the 0.15% AZE group, 27.9 days for patients in the 0.10% AZE group, and 27.5 days for patients in the placebo group. The mean number of doses taken by the three groups was similar: 53.4 doses in the 0.15% AZE group, 53.8 doses in the 0.10% AZE group, and 52.8 doses in the placebo group. The most commonly reported AEs (Table 2) in the 0.15% AZE group were nasal discomfort, occurring in 6.8% (3.6% in placebo and 0.10% AZE groups), and dysgeusia in the 0.10% AZE group, occurring in 5.6% (0.5% in the placebo and 4.7% in the 0.15% AZE groups) (Table 5).

**Table 5:** Treatment-emergent adverse effects with a frequency of ≥2% in any group (safety population).

Preferred term [*N* %]	0.15% AZE (*N* = 197)	0.10% AZE (*N* = 192)	Placebo (*N* = 192)
Nasal discomfort	7 (3.6)	13 (6.8)	7 (3.6)
Dysgeusia	11 (5.6)	9 (4.7)	1 (0.5)
Headache	4 (2.0)	1 (0.5)	6 (3.1)
Epistaxis	4 (2.0)	2 (1.0)	3 (1.6)
Sneezing	0	5 (2.6)	0
Other AEs of interest			
Fatigue	2 (1.0)	0	0
Somnolence	2 (1.0)	0	0

The frequency of dysgeusia was similar in the 0.15% AZE (*n* = 11 patients) and in the 0.10% AZE (*n* = 9 patients) groups. No subject had moderate or severe epistaxis or grade 2, 3, or 4 nasal irritation. The findings of direct visual nasal examination with 0.15% AZE and 0.10% AZE were similar to those seen with the placebo. No deaths or SAEs related to the study medication were reported.

## 5. Discussion

This study in PAR compared two doses of AZE (0.15% and 0.10%) with a placebo. The higher dose of AZE (0.15%) was tolerated similarly to the lower dose (0.10%) but it was the only dose that showed a statistically significant clinical improvement over the placebo. This study was only powered for comparison to the placebo, and therefore no significance vs. 0.10% AZE was expected in this study.

### 5.1. Strengths and limitations

It is difficult to show efficacy in PAR compared to the SAR study; for example, peak symptoms are less common in PAR than in SAR, and many patients have a rapid disappearance of symptoms when they suffer from intermittent rhinitis, making the differences between placebo and active treatment less marked. Symptoms in PAR patients such as nasal congestion, sneezing, blocked nose, and rhinorrhea tend to peak during the night and in the early morning (11). Hence, the mean severity of PAR symptoms is lower than that of SAR symptoms, making patients with PAR less sensitive to show differences in treatment.

In this study, patients receiving immunotherapy injections (antigen desensitization) had to be on a stable maintenance regimen for at least 30 days before the first study visit (adjustments to the regimen following a brief period of missed injections did not preclude participation). Patients receiving sublingual immunotherapy (SLIT) were excluded. A 6-month washout period was required after the last dose of sublingual immunotherapy. A total of five (2.6%), 11 (5.6%), and four (2.1%) subjects in the 0.15% AZE, 0.10% AZE, and placebo groups, respectively, received a stable maintenance regimen of immunotherapy injections during the study. Because these subjects received a stable maintenance regimen for years and the number of subjects receiving immunotherapy in the 0.15% AZE group was comparable to that in the placebo group and lower than that in the 0.10% AZE group, the inclusion of these subjects in the statistical analyses did not have a relevant impact on the demonstrated superiority of 0.15% AZE.

Guidelines recommend a longer treatment duration for PAR studies than for SAR studies (12). It is expected that variability in allergen exposure increases over time and therefore PAR studies are probably more affected than SAR studies in this respect. This also explains the higher placebo effect occasionally observed in PAR studies, which was also observed in the current study. Consequently, there is evidence that data from placebo-controlled PAR studies are more variable than those from SAR studies (13). Other authors have found the results of PAR studies to be more difficult to replicate than those of SAR studies (14).

### 5.2. Interpretation

Despite these difficulties, the primary objective was successfully met. The dosage of 0.15% AZE was statistically significant and superior to the placebo in relieving PAR symptoms. Studies have shown that AR patients use medications as needed, rapidly switch medications, resulting in poor control, and stop medications when symptoms are controlled (15). Hence, a medication with a rapid onset of action that is effective in targeting breakthrough symptoms is preferred. In a cross-sectional study by Price et al. (16), it was observed that the need for faster and more effective treatment was the primary reason for comedication in moderate/severe AR cases.

AZE has a distinctive bitter taste. Experiencing the bitter taste immediately after the use of the spray may be an indication of the wrong administration technique, causing unintended deposition of the formulation in the throat instead of the nasal cavities. Since such bitter taste sensations may interfere with the subject's adherence to the prescribed therapy, an improvement in the formulation is likely to improve patient compliance and, subsequently, treatment success. The present formulation (0.15% AZE) contains sucralose/sorbitol to mask the bitter taste of AZE. Adverse events reported by study subjects and documented by the investigator such as “bitter,” “sweet,” “strange,” “unpleasant,” and “metallic” were all coded as dysgeusia. Thus, studies using a parallel group design cannot show the effectiveness of taste masking.

The AEs occurring in the 0.10% AZE and 0.15% AZE groups were comparable, indicating that there was no dose-dependent increase in adverse effects. Somnolence and fatigue are two of the most common side effects associated with antihistamine usage. The present formulation of 0.15% AZE caused somnolence and fatigue in only 1.0% of the patients in the present study. The 0.15% AZE dosage effectively relieves not only nasal but also non-nasal symptoms (17). Nasal obstruction and ocular symptoms are associated with impaired work productivity in patients with allergic rhinitis (18). In the present study, 0.15% AZE provided significant improvements in nasal and non-nasal symptoms over placebo. Further, baseline rTNSS values were higher in the 0.15% AZE and 0.10% AZE groups compared to the placebo group. The 0.15% AZE dosage was still able to exhibit significant improvement over the placebo, showing its efficacy. Improvements in symptom scores in the 0.15% AZE group were consistently greater than those in the 0.10% AZE group, demonstrating dose-dependent symptom improvements.

### 5.3. Generalizability and application of the findings

The latest ARIA guidelines emphasize the onset of action as an important parameter for patients’ preferences for choosing the medication (19). Previous studies on SAR patients have shown an onset of action time of 30 min for 0.15% AZE (8). A recent chamber study with 0.15% AZE on PAR patients has shown its onset of action to be 30 min (20). AZE has also been shown to have a faster onset of action compared to mometasone in 450 SAR patients aged 18–65 years when tested in an environmental exposure chamber (21). A prospective study done on 240 patients with AZE and fluticasone (FLU) nasal sprays in a 1:1 ratio concluded that both intranasal AZE and FLU had comparable efficacy in symptom control in patients with AR (22). Intranasal corticosteroids are still the first-line therapy for the treatment of AR. A comparative study done between intranasal AZE and FLU has shown that FLU is superior to AZE in alleviating rhinorrhea. However, AZE showed comparable efficacy for all other nasal and ocular symptoms (4). As PAR patients require long-term medication compared to SAR patients, 0.15% AZE could be a safe option for children and patients with glaucoma and cataracts (22).

## 6. Conclusion

The present formulation of 0.15% AZE is safe and effective in relieving symptoms of PAR. It provides significant relief of both nasal and non-nasal symptoms. The maximum dose (two sprays per nostril twice daily) of 0.15% AZE did not reveal any safety concerns and was similar to that of 0.10% azelastine nasal spray.

## Data Availability

The original contributions presented in the study are included in the article/Supplementary Materials, further inquiries can be directed to the corresponding author.
